# *PRRT2* gene mutations associated with infantile convulsions induced by sucking and the genotype-phenotype correlation

**DOI:** 10.3389/fneur.2022.836048

**Published:** 2022-07-26

**Authors:** De-Tian Liu, Xue-Qing Tang, Rui-Ping Wan, Sheng Luo, Bao-Zhu Guan, Bin Li, Li-Hong Liu, Bing-Mei Li, Zhi-Gang Liu, Long-Shan Xie, Yong-Hong Yi

**Affiliations:** ^1^Key Laboratory of Neurogenetics and Channelopathies of Guangdong Province and the Ministry of Education of China, Institute of Neuroscience and Department of Neurology of the Second Affiliated Hospital of Guangzhou Medical University, Guangzhou, China; ^2^Department of Pediatrics, Foshan Women and Children Hospital Affiliated to Southern Medical University, Foshan, China; ^3^First People's Hospital of Foshan, Foshan, China

**Keywords:** *PRRT2*, infantile convulsion, seizures, sucking-induced, genotype-phenotype correlation

## Abstract

**Introduction:**

*PRRT2* is a major causative gene for self-limited familial neonatal-infantile epilepsy, paroxysmal kinesigenic dyskinesia, and paroxysmal kinesigenic dyskinesia with infantile convulsions. Voluntary movement trigger is prominent in adolescence and adulthood, but the triggers are unknown in infants.

**Methods:**

A gene panel designed for targeted next-generation sequencing (NGS) was used to screen genetic abnormalities in a cohort of 45 cases with infantile convulsions. The copy number variation was detected by a computational method based on the normalized depth of coverage and validated by a quantitative real-time polymerase chain reaction (RT-qPCR) method. The genotype-phenotype correlation of the *PRRT2* mutation gene was analyzed.

**Results:**

A *de novo* heterozygous *PRRT2* deletion was identified in a child who had infantile convulsions induced by vigorous sucking. Seizures happened during the change of feeding behavior from breast to formula, which led to hungry and vigorous sucking. Ictal electroencephalograms recorded seizures with focal origination, which provided direct evidence of epileptic seizures in infants with *PRRT2* mutations. Seizures stopped soon after the feeding behavior was changed by reducing feeding interval time and extending feeding duration. Data reanalysis on our previously reported cases with *PRRT2* mutations showed that six of 18 (33.3%) patients had infantile convulsions or infantile non-convulsion seizures during feeding. The mutations included two truncating mutations (c.579dupA/p.Glu194Argfs^*^6, and c.649dupC/p.Arg217Profs^*^8) that were identified in each of the three affected individuals.

**Conclusions:**

This study suggests that feeding, especially vigorous sucking, is potentially a trigger and highlights the significance of feeding behavior in preventing seizures in infants with *PRRT2* mutations. Identification of *PRRT2* haploinsufficiency mutations in the patients with infantile convulsions induced by sucking suggested a potential genotype-phenotype correlation.

## Introduction

The *PRRT2* gene (OMIM: 614386) encodes a transmembrane protein containing a proline-rich domain in the N-terminal half and being involved in synaptic transmission (1). *PRRT2*-associated diseases are mainly paroxysmal kinesigenic dyskinesia (PKD) and paroxysmal hypnogenic dyskinesia (PHD) in adults and self-limited familial neonatal-infantile epilepsy or infantile convulsion and choreoathetosis (ICCA) in infants. Infantile convulsion (IC) is the most common feature in early life among the wide spectrum of *PRRT2* phenotypes (2). A previous study demonstrated that self-limited familial neonatal-infantile epilepsy accounted for 41.7%, PKD accounted for 38.7%, and ICCA accounted for 14.3% of the *PRRT2*-associated phenotypes (3). The *PRRT2* mutations previously reported included missense, non-sense, splicing, deletions, insertions, indels, and complex rearrangement; and the majority are truncating mutations that lead to haploinsufficiency. Voluntary movement triggering is a prominent feature in patients of adolescence and adulthood, which is highlighted in the diagnostic criteria for PKD (2). However, the triggers of attacks in early childhood were unknown. We previously observed several patients with *PRRT2* mutations that had attacks during feeding, suggesting sucking as a possible kinesigenic trigger (4). In this study, we screened a cohort of 45 cases with infant seizures and identified a *PRRT2* heterozygous deletion mutation in a child with frequent seizures induced by vigorous sucking and became seizure-free after changing feeding behavior. We analyzed the clinical and genetic features of the cases who had attacks during feeding and found that their *PRRT2* mutations were all truncating or deletion mutations that would lead to haploinsufficiency, which suggested a potential genotype-phenotype correlation and clinical significance.

## Materials and methods

### Patients

A total of 45 patients with infant convulsion were recruited from the Epilepsy Center of the Second Affiliated Hospital of Guangzhou Medical University and the First People's Hospital of Foshan. Epileptic seizures and epilepsy syndromes were diagnosed and classified according to the criteria of the Commission on Classification and Terminology of the International League Against Epilepsy (1981, 1989, 2001, 2010, and 2017). The collected clinical data included detailed semiology, onset age, seizure type, the evolution of disorder and frequency, family history, response to anti-seizure medications, general and neurological examination, and brain magnetic resonance imaging (MRI). Long-term video-EEG monitoring records that included open-close eyes test, hyperventilation, intermittent photic stimulation, and sleep recording were obtained.

This study complied with the principles of the International Committee of Medical Journal Editors about patient consent for research or participation. Written informed consent was obtained from the individuals or legal guardians. Ethical approval had been obtained from the Ethics Committee of the Second Affiliated Hospital of Guangzhou Medical University.

### Targeted sequencing and filtering

Peripheral blood samples were collected from the probands, their parents, and other family members if available. Genomic DNA (gDNA) was extracted using the Qiagen Flexi Gene DNA Kit (Qiagen, Hilden, Germany). The *PRRT2* mutations in the early six cases in our previous study were identified by direct Sanger sequencing, targeting the coding regions (4). A custom-targeted NGS gene panel was performed in recent years to screen gene abnormalities associated with episodic disorders (5), including *PRRT2, CHRNA4, KCNA1, KCNQ2, KCNMA1, PNKD, SCN2A*, and *SLC2A1* genes that are associated with BFIS and PKD. Trios-based exome paired-end sequencing was performed on the Illumina Nextseq 500 platform by capture probe NeuMet_V1. The mean read depth for each sample was 208-fold. The raw data were aligned to the reference genome (GRCh37/hg19). Single nucleotide variants (SNVs) and insertions/deletions (indels) filtering were performed by using the Genome Analysis Toolkit (6). Stepwise filtering was applied to the derived candidate causative variants as we previously described (7). The copy number variations (CNVs) were performed by a computational method (CNVkit) based on the normalized depth of coverage, which used both the targeted reads and the non-specifically captured off-target reads to infer copy number (8). Sanger sequencing was applied to confirm the candidate variants.

### Quantitative real-time PCR (QPCR) analysis

The qPCR was used to verify the heterozygous *PRRT2* deletion at the gDNA level. The parents were regarded as healthy controls. Primers F1 (5′-GGATGCAGAGGGAGTGGAATG-3′) and R1 (5′-CCCAGCACCCCAAAATCAAC-3′) were designed to amplify the fore part of *PRRT2*, primers F2 (5′-GAAGGCACCCAGAAACCTCG-3′) and R2 (5′-ACAGCATAAGCGAAGGCCAC-3′) were designed to amplify the middle part of *PRRT2*, and primers F3 (5′-AACACCTCCCCAACTCTGCG-3′) and R3 (5′-CCAGGGTGGAGGTCCAGAGAAT-3′) were designed to amplify the whole length of *PRRT2*. The total volume of each sample reaction was 10 μl, including 1.5 μl (15 ng gDNA), 1 μl forward primer (2 μM), 1 μl reverse primer (2 μM),.2 μl ROX Reference Dye (50×), 5 μl SYBR Premix Ex Taq (2×), and 1.3 μl double distilled water. Each sample was run in triplicate on a 7300 Realtime PCR System (Applied Biosystems) by using the SYBR^®^ Premix Ex Taq™ Reagent (Takara). The expression of *POLR2A* as a reference gene was used for data normalization. Relative ratios of the gene expression were calculated by formula: r = 2^−ΔΔCt^ with ΔΔCt = (Ct_*PRRT2*_ – Ct_*POLR2A*_) _ind tested_ - (Ct_*PRRT2*_ – Ct_*POLR2A*_) _ind ref_. Values were calculated about the father and arbitrarily attributed to the value of 1.0. SPSS 19.0 software (Chicago, USA) was used for statistical analysis.

## Results

**Figure 1:**
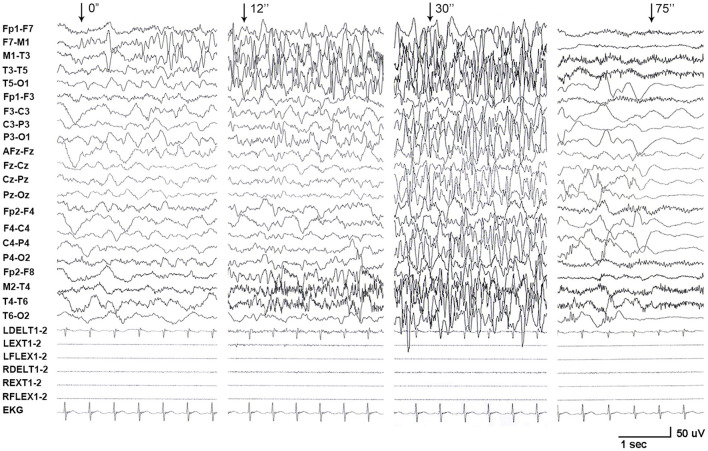
EEG recordings from patient with *PRRT2* variants. A long-term video electroencephalogram recorded a seizure that started with spikes originating from the left frontal lobe and gradually generalized, lasting for 75 s, followed by electrical suppression.

**Figure 2:**
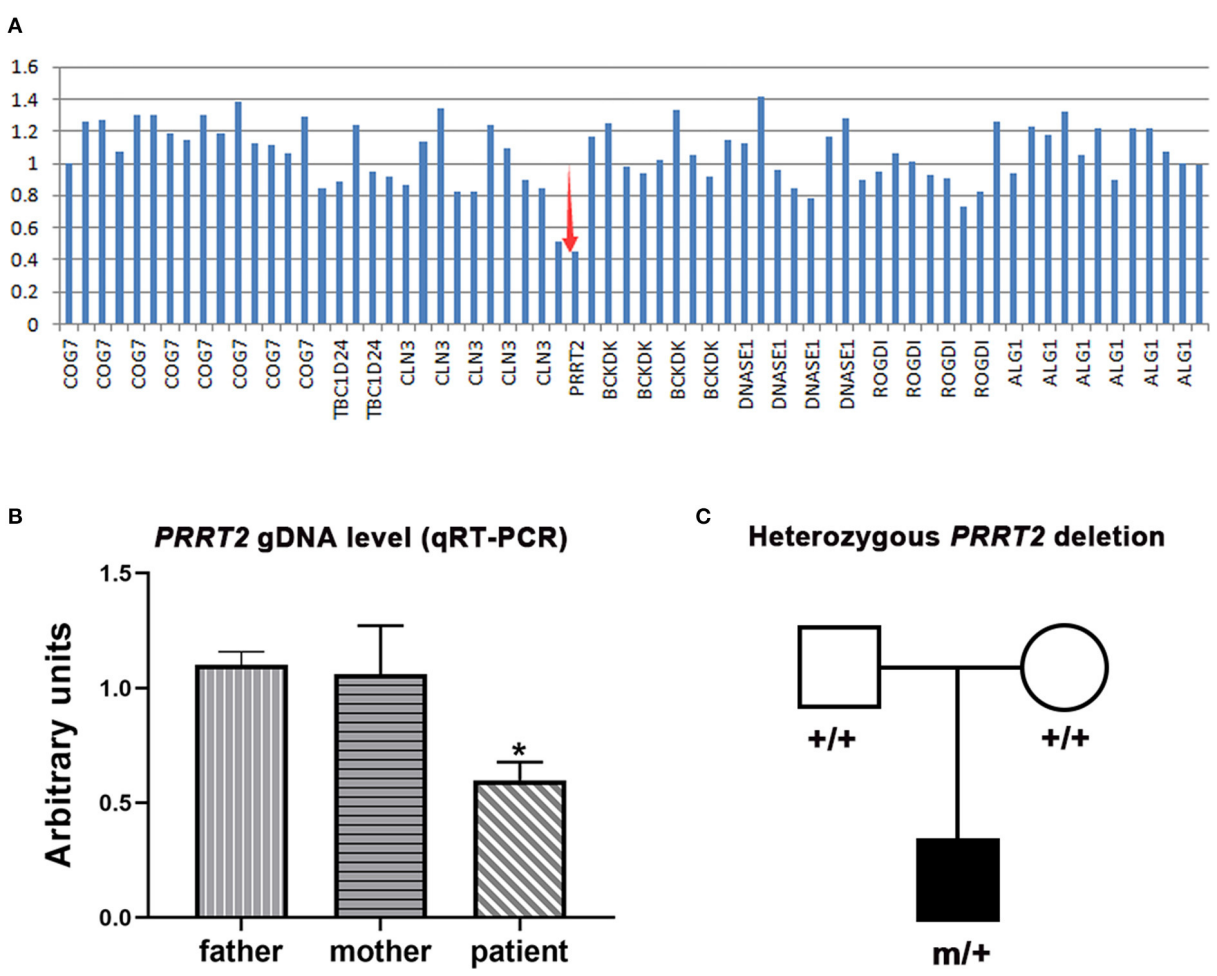
Genetic data of the cases with *PRRT2* variants. **(A)** Copy number variations analysis showed a heterozygous *PRRT2* deletion (red arrows). **(B)** Relative quantification of genomic DNA (gDNA) confirmed the deletion. The patient presented a reduced *PRRT2* gDNA level (half of the level of his parents). Error bar, mean ± SEM, *n* = 3; *p < 0.05, vs. his father and mother. **(C)** Pedigree of the family, showing the heterozygous *PRRT2* deletion was a *de novo* mutation.

One *de novo PRRT2* mutation was identified in a child with infant seizures. The child was a full-term boy who was born to healthy non-consanguineous parents. His early development was normal. The seizures began at the age of 4 months when breastfeeding was switched to formula feeding. He presented his first convulsion when he started sucking eagerly and vigorously. The seizure began with a deviation of head and eyes to the right, then cyanosis, and followed by the loss of consciousness and generalized hypertonic lasting about 1 min. The attacks repeated 2–10 times per day and mostly occurred during vigorous sucking. The ictal EEGs recorded a seizure that started with spikes from the left frontal lobe and gradually generalized, lasting for 75 s, followed by electrical suppression (Figure 1). Interictal EEGs revealed low voltage spikes and waves in the left frontal lobe with a normal background. Brain MRI was normal. Blood routine and biochemical tests were normal. Valproate (16 mg/kg/d) was applied on the third day after onset but did not stop the seizures. Vigorous sucking was subsequently avoided by shortening interval time, extending the duration of feeding, and adding the rice paste. The child was seizure-free since then (followed-up for 2 years until now). EEG became normal in the third month after being seizure-free. Valproate was discontinued after a year. The neurodevelopment was normal at 3 years of follow-up. Using the gene panel, we failed to find any pathogenic point mutations in this child. Heterozygous *PRRT2* gene deletion was identified by the computational method of CNVs (Figure 2A) and validated by the qPCR method (Figure 2B). The mutation was a heterozygous whole deletion of one allele of the *PRRT2* gene, which led to haploinsufficiency. Co-segregation analysis showed that the mutation was a *de novo* mutation (Figure 2C).

We reanalyzed the data from the cohort of 34 individuals with *PRRT2* mutations (Figure 2 in that article) (4). Six patients were found to have experienced feeding-associated infantile convulsions or infantile non-convulsion seizures. The clinical and genetic features of the patients are summarized in Table 1. All patients had heterozygous deleterious mutations, including two truncating mutations, c.579dupA/p.Glu194Argfs^*^6, and c.649dupC/p.Arg217Profs^*^8, that were identified in each of the three affected individuals. Mutation c.649dupC/p.Arg217Profs^*^8 has previously been reported as a hot-spot mutation (3). Except for the deletion mutation that was *de novo*, the others were of familial inheritance. The average age at onset was 6.1 ± 2.1 months (range 4–10 months). Five patients exhibited classical convulsions. Two patients presented non-convulsive seizures that manifested as a stop of suction, loss of responses, and then cyanosis without a generalized tonic seizure. All patients had normal EEG backgrounds. Three of them had focal (frontal/ temporal) spikes and sharp or slow waves. Six were in remission before 12 months and one was in remission at age of 3 years old. Five patients developed paroxysmal disorders or paroxysmal dyskinesias later in adolescence.

**Table 1:** Genetic and clinical characteristics of patients with feeding-induced infantile convulsion/non-convulsion seizure caused by *PRRT2* mutations.

**Mutation**	**Inheritance/sex**	**Seizure**	**Onset age**	**Frequency**	**Duration**	**Remission age**	**EEG**	**ASMs**	**Evolution/**
									**onset age**
c.579dupA/p.Glu194Argfs*6	Familial/Male	IC	5 mon.	2–3 times/day	2 min.	12 mon.	Normal	None	PKD/7 yr.
c.579dupA/p.Glu194Argfs*6	Familial/Female	IC	5 mon.	0–1 time/week	20 sec.	12 mon.	Normal	None	PKD/13 yr.
c.579dupA/p.Glu194Argfs*6	Familial/Female	IC	5 mon.	1–2 times/day	20 sec.	12 mon.	Normal	None	PKD/5 yr.
c.649dupC/p.Arg217Profs*8	Familial/Male	IC	6 mon.	2–3 times/day	1 min.	6 mon.	Normal	PB	PKD/13 yr.
c.649dupC/p.Arg217Profs*8	Familial/Female	INCS-IC	8 mon.	4 times-once	1–2 min.	9 mon.	RTSW	VPA	NA
c.649dupC/p.Arg217Profs*8	Familial/Male	INCS	10 mon.	0–8/day	~10 sec.	3 yr.	RF-Sharp	PB	PD/9 yr.
Entire *PRRT2* heterozygote deletion	*De novo*/Male	IC	4 mon.	2–10 times/day	1 min.	5 mon.	Spikes	VPA	Follow-up

ASMs, anti-seizure medications; IC, infantile convulsion; INCS, infantile non-convulsion seizure; NA, no not available; PB, phenobarbital; PD, Paroxysmal dyskinesias; PKD, paroxysmal kinesigenic dyskinesia; RF, right frontal; RTSW, right temporal slow wave; VPA, Valproate.

## Discussion and conclusions

The *PRRT2* has been identified as the most common causative gene of PKD (9) and other paroxysmal disorders. Loss of function of the gene results in a delay of neuronal migration and a marked decrease in synaptic density, which were suggested to be the mechanism underlying *PRRT2*-associated disease (1, 10). Paroxysmal movement attack induced by triggering is a prominent feature of the disorders associated with *PRRT2* mutations. In this study, we reported seven cases with *PRRT2* mutations who presented feeding-induced infantile seizures. Ictal EEGs recorded a seizure with focal origination in the case induced by vigorous sucking. This study suggests feeding, especially sucking, as a potential trigger factor. The seven cases had deletion or truncating mutations that resulted in haploinsufficiency of the *PRRT2* gene, suggesting a potential genotype-phenotype correlation. To date, 155 *PRRT2* mutations have been identified in patients with episodic disorders (Human Gene Mutation Database, www.hgmd.cf.ac.uk/ac/index.php). Mutations of haploinsufficiency accounted for 60.6%, including truncating, deletion, insertion, and splice-site mutations. The other mutations were missenses. The hot spot mutation c.649dupC/p.Arg217Profs^*^8 alone even contributed to 73.8% of PKD/IC etiology in a previous study (3). In our early cohort, six of the 18 (33.3%) patients with *PRRT2* variant had IC and infantile no-convulsion seizures during feeding (4). Therefore, feeding potentially triggers movement in infants with *PRRT2* mutations, especially in those with mutations of haploinsufficiency.

Previously, it was not determined whether the infant convulsion in patients with *PRRT2* mutations was epileptic seizures in some cases. Marini et al. (11) reported a recorded focal seizure in a child with *PRRT2* mutation. The reported case and the case presented in this study provided direct evidence of epileptic seizures in infants with *PRRT2* mutations. Therefore, attention should be paid to triggers in infants with convulsions or seizures. The child in this study got seizure-free since the change of feeding behavior, highlighting the significance of feeding behavior in the early prevention of epileptic seizures and the possible secondary brain injury due to frequent seizures. Vigorous sucking behavior, such as feeding in hunger, should be avoided by maneuvers like reducing feeding interval time and extending feeding duration in infants with *PRRT2* mutations. This case also suggests sucking-induced seizures as a reminder of *PRRT2* mutations and further genetic tests. Although some patients do not have seizures after a year or two, frequent seizures are harmful. It is crucial to identify the trigger and use appropriate antiepileptic drugs, such as carbamazepine, to stop the seizures.

In conclusion, we identified a *de novo* heterozygous *PRRT2* deletion in a patient who presented infantile seizures induced by sucking. Data reanalysis on our previously reported cases with *PRRT2* mutations showed that 33.3% of patients had infantile convulsions or infantile no-convulsion seizures during feeding. This study suggests sucking as a potential trigger movement in infants with *PRRT2* mutations, highlighting the significance of feeding behavior in the early prevention of epileptic seizures. All seven patients had *PRRT2* mutations of haploinsufficiency, suggesting a potential genotype-phenotype correlation.

## Data availability statement

The datasets presented in this study can be found in online repositories. The names of the repository/repositories and accession number(s) can be found at: NCBI - PRJNA833568.

## Ethics statement

All procedures performed were in accordance with the ethical standards of the institutional committee. The present study was approved by the Ethics Committee of the Second Affiliated Hospital of Guangzhou Medical University.

## Author contributions

Substantial contributions to conception and design, acquisition of data, or analysis and interpretation of data were made by SL, B-ZG, BL, L-HL, B-ML, and Z-GL. Drafting the article or revising it critically for important intellectual content were made by Y-HY, L-SX, D-TL, X-QT, and R-PW. Final approval of the version to be published was done by all authors.

## Funding

This work was supported by grants from the National Natural Science Foundation of China (Grant No. 81870903). The funders had no role in study design, data collection, and analysis or in the decision to publish and neither in the preparation of the manuscript.

## Conflict of interest

The authors declare that the research was conducted in the absence of any commercial or financial relationships that could be construed as a potential conflict of interest.

## Publisher's note

All claims expressed in this article are solely those of the authors and do not necessarily represent those of their affiliated organizations, or those of the publisher, the editors and the reviewers. Any product that may be evaluated in this article, or claim that may be made by its manufacturer, is not guaranteed or endorsed by the publisher.

## Abbreviations

PKD: paroxysmal kinesigenic dyskinesia
PHD: paroxysmal hypnogenic dyskinesia
ICCA: infantile convulsion and choreoathetosis
IC: infantile convulsion
EEG: electroencephalogram
MRI: brain magnetic resonance imaging
CNVs: copy number variations
qPCR: quantitative real-time-polymerase chain reaction.

## References

[1] Liu YT Nian FS Chou WJ Tai CY Kwan SY Chen C. PRRT2 mutations lead to neuronal dysfunction and neurodevelopmental defects. Oncotarget. (2016) 7:39184–96. 10.18632/oncotarget.9258 27172900 PMC5129924

[2] Bruno MK Hallett M Gwinn-Hardy K Sorensen B Considine E Tucker S. Clinical evaluation of idiopathic paroxysmal kinesigenic dyskinesia: new diagnostic criteria. Neurology. (2004) 63:2280–7. 10.1212/01.WNL.0000147298.05983.50 15623687

[3] Ebrahimi-Fakhari D Saffari A Westenberger A Klein C. The evolving spectrum of PRRT2-associated paroxysmal diseases. Brain. (2015) 138(Pt. 12):3476–95. 10.1093/brain/awv317 26598493

[4] Liu XR Wu M He N Meng H Wen L Wang JL. Novel PRRT2 mutations in paroxysmal dyskinesia patients with variant inheritance and phenotypes. Genes Brain Behav. (2013) 12:234–40. 10.1111/gbb.12008 23190448

[5] Zhou P He N Zhang JW Lin ZJ Wang J Yan LM. Novel mutations and phenotypes of epilepsy-associated genes in epileptic encephalopathies. Genes Brain Behav. (2018) 17:e12456. 10.1111/gbb.12456 29314583

[6] DePristo MA Banks E Poplin R Garimella KV Maguire JR Hartl C. A framework for variation discovery and genotyping using next-generation DNA sequencing data. Nat Genet. (2011) 43:491–8. 10.1038/ng.806 21478889 PMC3083463

[7] Chen ZR Liu DT Meng H Liu L Bian WJ Liu XR. Homozygous missense TPP1 mutation associated with mild late infantile neuronal ceroid lipofuscinosis and the genotype-phenotype correlation. Seizure. (2019) 69:180–5. 10.1016/j.seizure.2018.08.027 31059981

[8] Talevich E Shain AH Botton T Bastian BC. CNVkit: genome-wide copy number detection and visualization from targeted DNA sequencing. PLoS Comput Biol. (2016) 12:e1004873. 10.1371/journal.pcbi.1004873 27100738 PMC4839673

[9] Chen WJ Lin Y Xiong ZQ Wei W Ni W Tan GH. Exome sequencing identifies truncating mutations in PRRT2 that cause paroxysmal kinesigenic dyskinesia. Nat Genet. (2011) 43:1252–5. 10.1038/ng.1008 22101681

[10] Tan G-H Liu Y-Y Wang L Li K Zhang Z-Q Li H-F. PRRT2 deficiency induces paroxysmal kinesigenic dyskinesia by regulating synaptic transmission in cerebellum. Cell Res. (2017) 28:90–110. 10.1038/cr.2017.128 29056747 PMC5752836

[11] Marini C Conti V Mei D Battaglia D Lettori D Losito E. PRRT2 mutations in familial infantile seizures, paroxysmal dyskinesia, and hemiplegic migraine. Neurology. (2012) 79:2109–14. 10.1212/WNL.0b013e3182752ca2 23077026 PMC3511926

